# Supplementary material for: Estimating tumor mutational burden from RNA-sequencing without a matched-normal sample
Source: Nat Commun. 2022 Jun 2;13:3092. doi: 10.1038/s41467-022-30753-2 (PMC9163107; doi:10.1038/s41467-022-30753-2)
Supplement: Supplementary file 1 — Supplementary Information [file 41467_2022_30753_MOESM1_ESM.pdf]

Supplementary Material

Estimating tumor mutational burden from RNA-sequencing without matched-normal

Supplementary Figures:

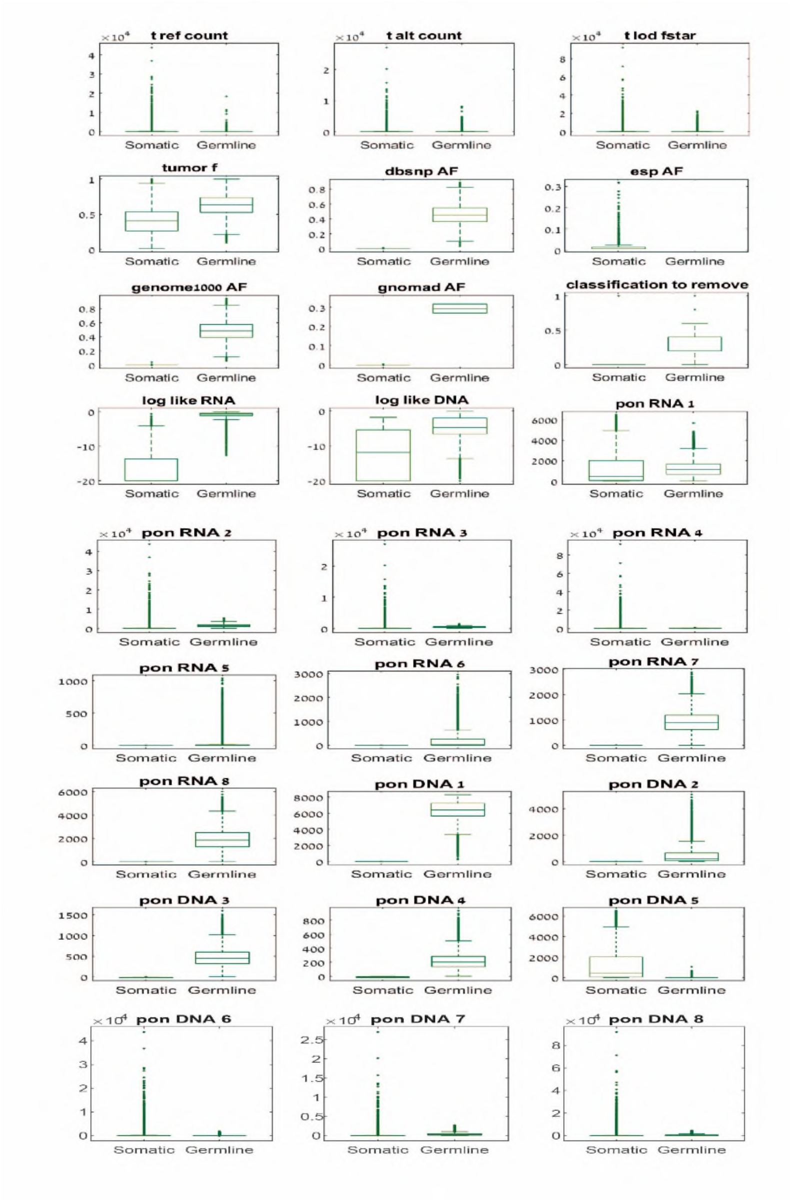

Supplementary Figure 1: Feature comparison between somatic and germline variants (n=11,843,749). Boxplots describing the different feature values between somatic and germline variants. Box plots show median, 25th, and 75th percentiles. The whiskers extend to the most extreme data points not considered outliers, and the outliers are represented as dots.

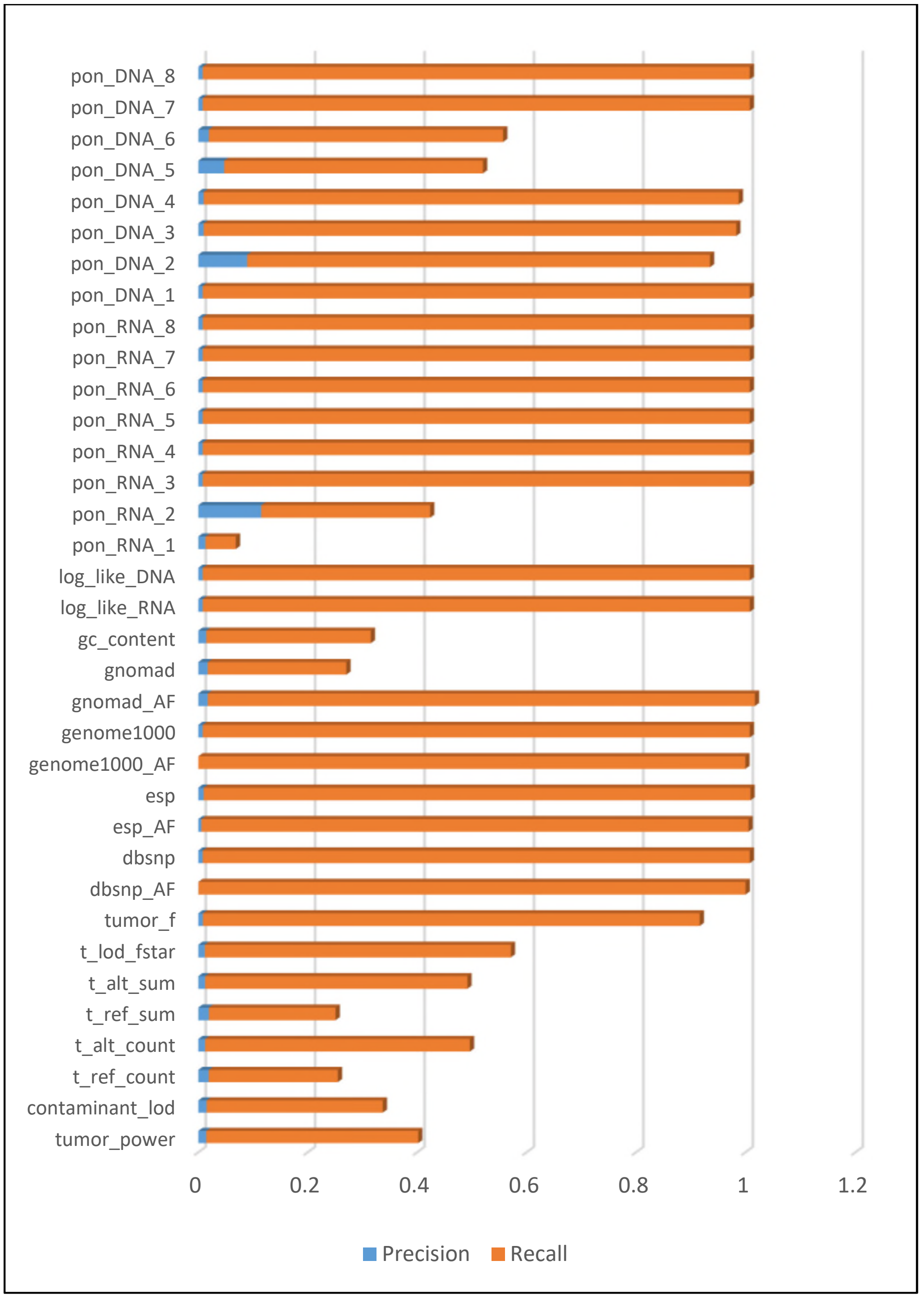

Supplementary Figure 2: Feature precision and recall values. Best precision and recall achieved for each feature, using the threshold providing the optimal F1-score when calculated across a range of thresholds.

file.

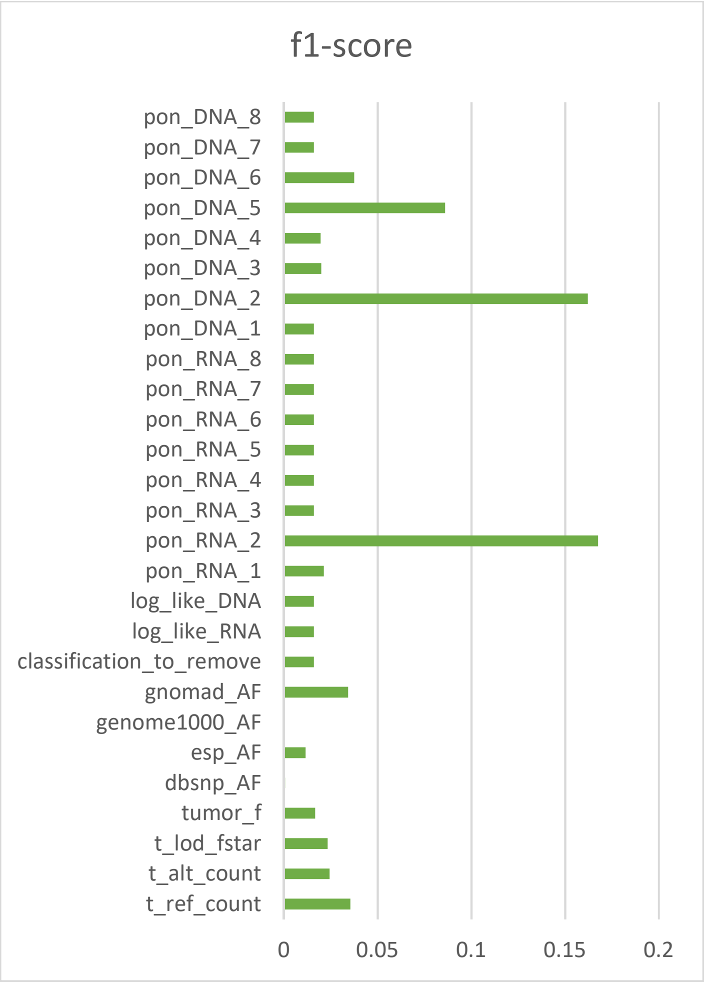

Supplementary Figure 3: Feature F1 scores. Best F1-score for each feature obtained by calculating the precision/recall across a range of thresholds.

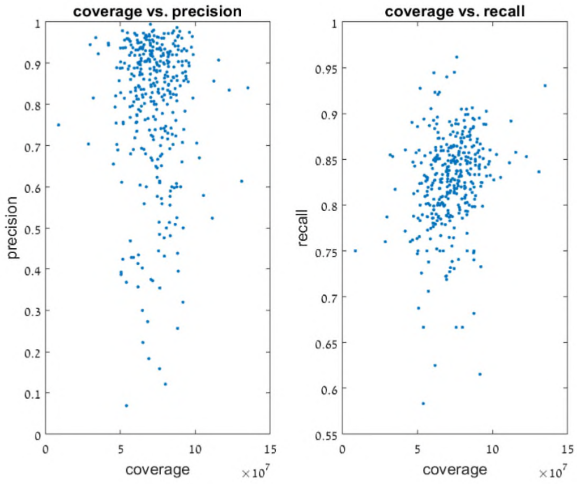

Supplementary Figure 4: Sequence coverage correlations. Spearman correlation between sequence coverage and sample precision (left) and sample recall (right). Source data are provided as a Source Data file.

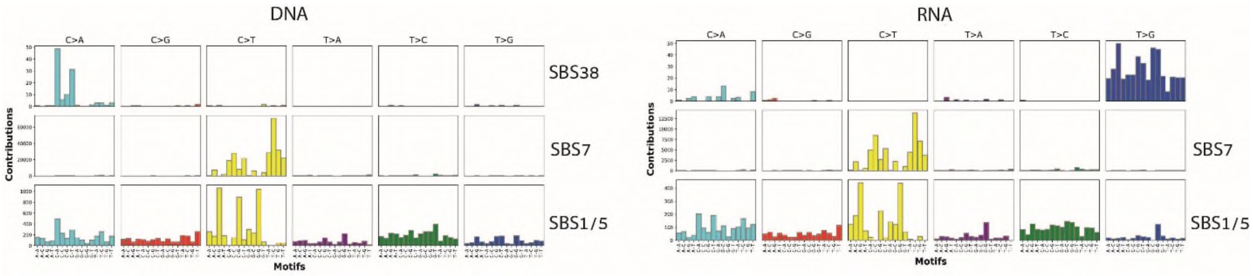

Supplementary Figure 5: Melanoma signature analysis. Mutational signatures[1] identified in the DNA and RNA of the melanoma dataset. SBS7 (cosine similarity = 0.97 and 0.95 in DNA and RNA, respectively); a combination of SBS1 and SBS5 (cosine similarity = 0.76/0.71 and 0.66/0.71 in DNA and RNA, respectively). In addition, SBS38 is identified in the DNA and in the predicted RNA (Figure 2a, cosine similarity = 0.98), and a signature enriched with T>G mutations is identified in the RNA, similar to the one identified based on the predicted set of mutations using RNA alone (Figure 2a). Source data are provided as a Source Data file.

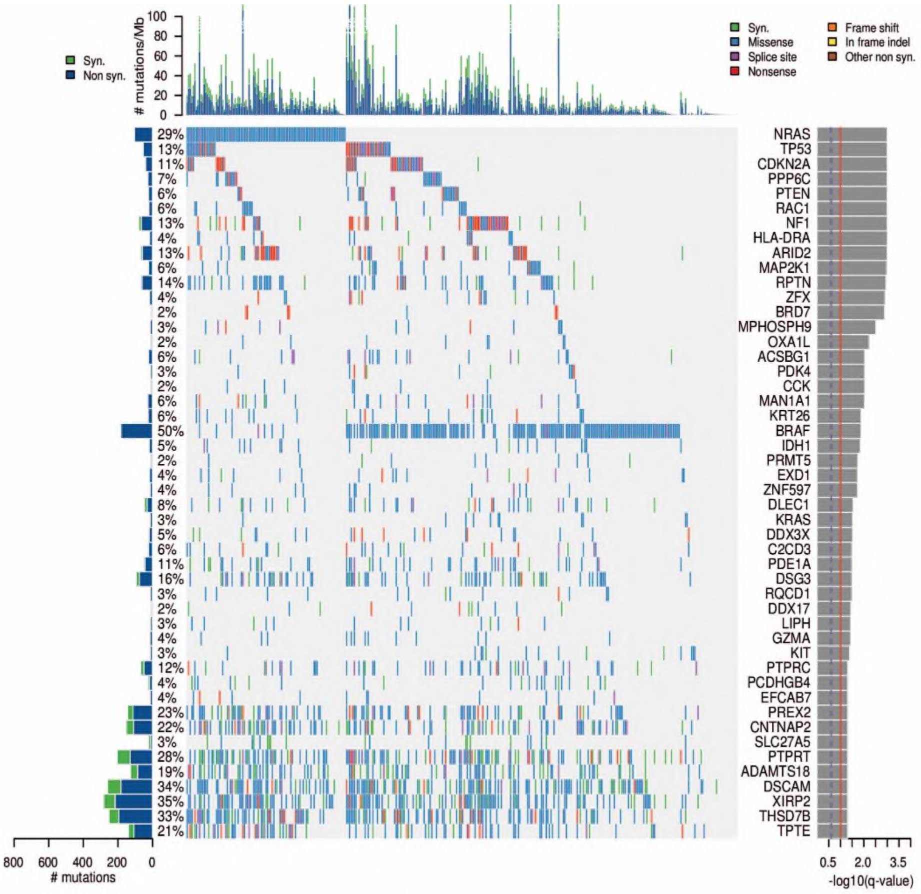

Supplementary Figure 6: Melanoma significantly mutated genes. Co-mutation plot based on the set of somatic mutations detected using tumor and matched-normal DNA from the melanoma dataset. Overall frequencies, allele fractions, and significance levels of candidate cancer genes (Q < 0.05) identified by MutSig2CV[2] are shown. Source data are provided as a Source Data file.

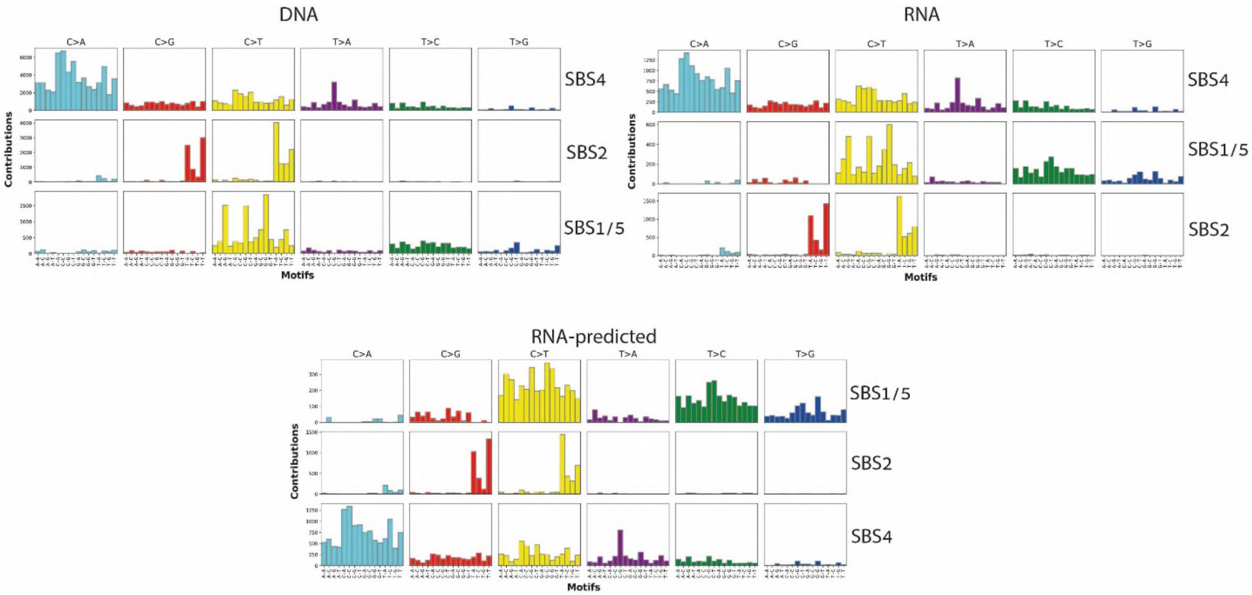

Supplementary Figure 7: Lung signature analysis. 3 mutational signatures[1] are identified in the lung dataset, using DNA, RNA and predicted RNA mutations calls. SBS4 (cosine similarity = 0.94, 0.91 and 0.92 in the 3 cases, respectively); SBS2 (cosine similarity = 0.76, 0.71 and 0.69 in the 3 cases, respectively); and a combination of SBS1 and SBS5 (cosine similarity = 0.84/0.78, 0.72/0.81 and 0.48/0.88 in the 3 cases, respectively). Source data are provided as a Source Data file.

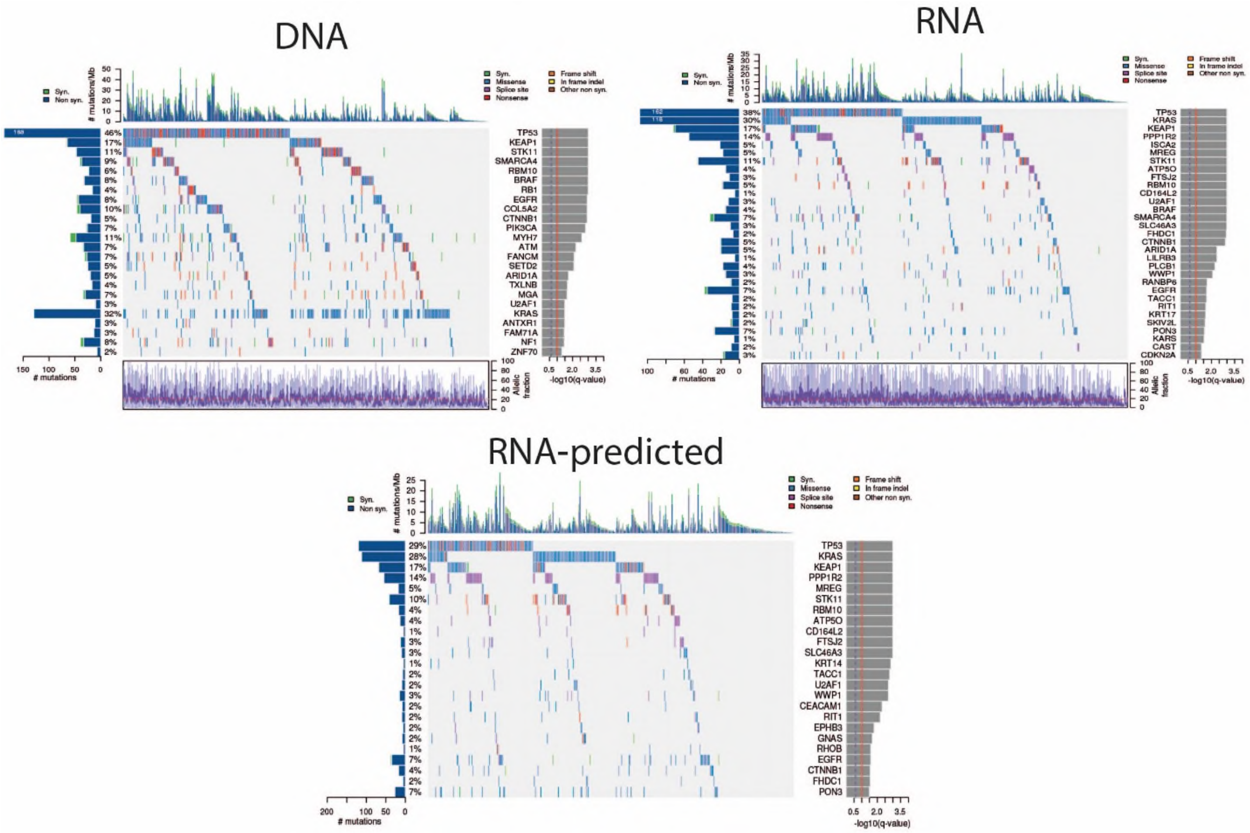

Supplementary Figure 8: Lung significantly mutated genes. Co-mutation plot based on the set of somatic mutations detected using tumor and matched-normal DNA, tumor RNA and matched-normal DNA and tumor RNA alone of the lung dataset. Overall frequencies, allele fractions, and significance levels of candidate cancer genes ($Q < 0.05$) identified by MutSig2CV[2] are shown. Source data are provided as a Source Data file.

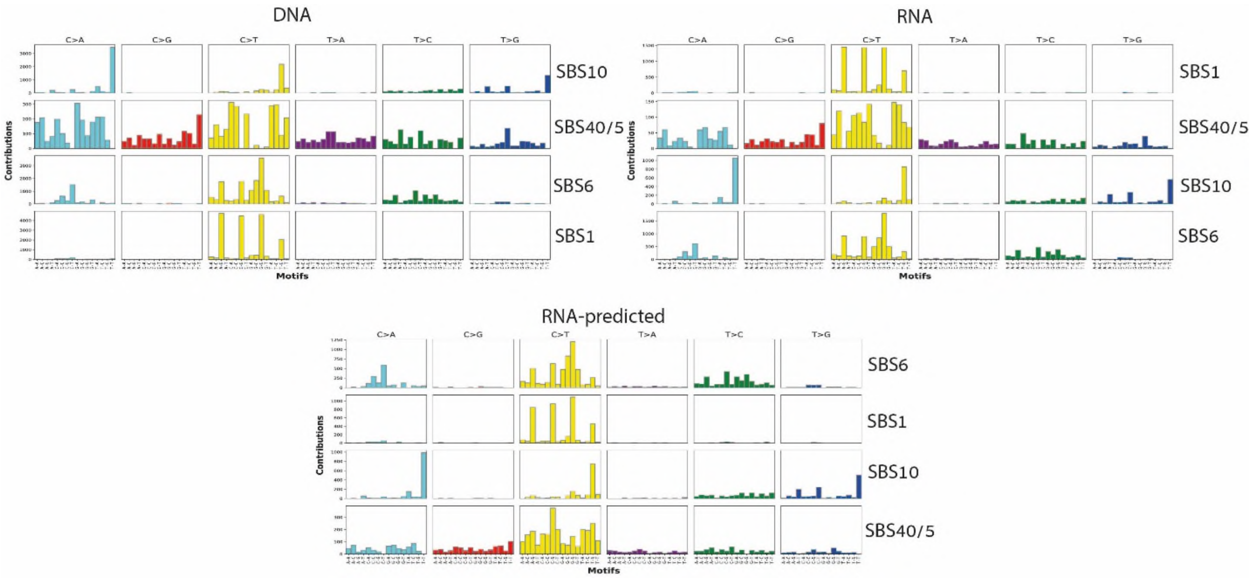

Supplementary Figure 9: Colon signature analysis. 4 mutational signatures[1] are identified in the colon dataset, using DNA, RNA and predicted RNA mutations calls. SBS1 (cosine similarity = 0.99, 0.99 and 0.98 in the 3 cases, respectively); SBS6 (cosine similarity = 0.88, 0.9 and 0.84 in the 3 cases, respectively); SBS10 (cosine similarity = 0.8, 0.7 and 0.72 in the 3 cases, respectively); and a combination of SBS40 and SBS5 (cosine similarity = 0.86/0.65, 0.81/0.74 and 0.67/0.83 in the 3 cases, respectively). Source data are provided as a Source Data file.

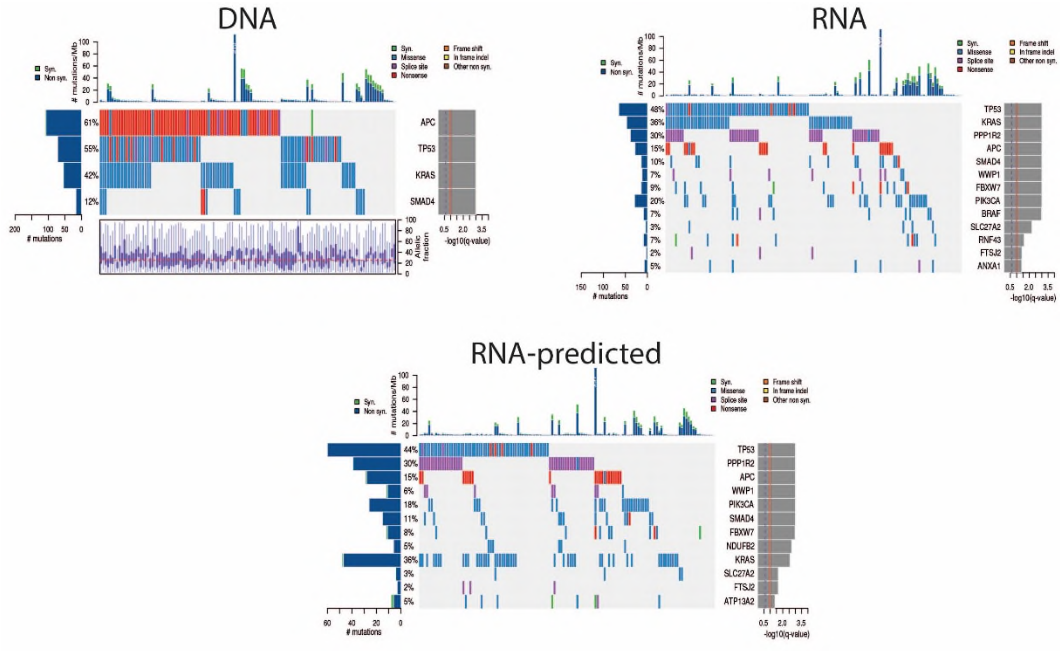

Supplementary Figure 10: Colon significantly mutated genes. Co-mutation plot based on the set of somatic mutations detected using tumor and matched-normal DNA, tumor RNA and matched-normal DNA and tumor RNA alone in the colon dataset. Overall frequencies, allele fractions, and significance levels of candidate cancer genes ($Q < 0.05$) identified by MutSig2CV[2] are shown. Source data are provided as a Source Data file.

Supplementary Note 1:

Supplementary Code

Pseudocode

```
for i=1:5
        load train_i
        run RandomForest
        save model_i
end
compute precision and recall (train)

for i=1:5
        load test
        apply model i
end
take majority vote
compute precision and recall (test)
```

References

1. Kim, J. *et al.* Somatic ERCC2 mutations are associated with a distinct genomic signature in urothelial tumors. *Nat Genet* **48**, 600–606 (2016).

2. Lawrence, M., Stojanov, P. & Mermel, C. Discovery and saturation analysis of cancer genes across 21 tumour types. *Nature* **505**, 495–501.
