# Supplementary material for: Estimating tumor mutational burden from RNA-sequencing without a matched-normal sample
Source: Nat Commun. 2022 Jun 2;13:3092. doi: 10.1038/s41467-022-30753-2 (PMC9163107; doi:10.1038/s41467-022-30753-2)
Supplement: Supplementary file 2 — Description to Additional Supplementary Information [file 41467_2022_30753_MOESM2_ESM.pdf]

**Description of Additional Supplementary Information**

Supplementary Code: Code for running the ML pipeline

Supplementary Data: Supplementary raw data for all figures
