# Supplementary material for: Estimating tumor mutational burden from RNA-sequencing without a matched-normal sample
Source: Nat Commun. 2022 Jun 2;13:3092. doi: 10.1038/s41467-022-30753-2 (PMC9163107; doi:10.1038/s41467-022-30753-2)
Supplement: Supplementary file 5 — Reporting Summary [file 41467_2022_30753_MOESM5_ESM.pdf]

nature portfolio

Corresponding author(s): Keren Yizhak

Last updated by author(s): 20/04/2022

Reporting Summary

Nature Portfolio wishes to improve the reproducibility of the work that we publish. This form provides structure for consistency and transparency in reporting. For further information on Nature Portfolio policies, see our Editorial Policies and the Editorial Policy Checklist.

Statistics

For all statistical analyses, confirm that the following items are present in the figure legend, table legend, main text, or Methods section.

n/a	Confirmed	
☐	☒	The exact sample size (*n*) for each experimental group/condition, given as a discrete number and unit of measurement
☐	☒	A statement on whether measurements were taken from distinct samples or whether the same sample was measured repeatedly
☐	☒	The statistical test(s) used AND whether they are one- or two-sided<br>*Only common tests should be described solely by name; describe more complex techniques in the Methods section.*
☐	☒	A description of all covariates tested
☐	☒	A description of any assumptions or corrections, such as tests of normality and adjustment for multiple comparisons
☐	☒	A full description of the statistical parameters including central tendency (e.g. means) or other basic estimates (e.g. regression coefficient) AND variation (e.g. standard deviation) or associated estimates of uncertainty (e.g. confidence intervals)
☐	☒	For null hypothesis testing, the test statistic (e.g. *F*, *t*, *r*) with confidence intervals, effect sizes, degrees of freedom and *P* value noted<br>*Give P values as exact values whenever suitable.*
☒	☐	For Bayesian analysis, information on the choice of priors and Markov chain Monte Carlo settings
☒	☐	For hierarchical and complex designs, identification of the appropriate level for tests and full reporting of outcomes
☒	☐	Estimates of effect sizes (e.g. Cohen's *d*, Pearson's *r*), indicating how they were calculated

Our web collection on statistics for biologists contains articles on many of the points above.

Software and code

Policy information about availability of computer code

Data collection	All data used in this study was downloaded manually and processed by the softwares described below.
Data analysis	Data analysis was done using:<br>(1) Terra, Beta version, a cloud-based tool for running analyses at scale;<br>(2) Python v3.9.1, for running the ML pipeline;<br>(3) Matlab 2018b, for statistical testing and for preparing figures.<br>(4) STAR v2.4.1 for aligning raw sequencing reads<br>(5) MuTect v1.1.6 for calling somatic mutations<br>(6) NovoAlign v2 for the realignement filter

For manuscripts utilizing custom algorithms or software that are central to the research but not yet described in published literature, software must be made available to editors and reviewers. We strongly encourage code deposition in a community repository (e.g. GitHub). See the Nature Portfolio guidelines for submitting code & software for further information.

Data

Policy information about availability of data

All manuscripts must include a data availability statement. This statement should provide the following information, where applicable:

- Accession codes, unique identifiers, or web links for publicly available datasets
- A description of any restrictions on data availability
- For clinical datasets or third party data, please ensure that the statement adheres to our policy

Access to TCGA raw sequencing data (DNA and RNA, phs000178) was obtained via dbGap authorization [https://www.ncbi.nlm.nih.gov/projects/gap/cgi-bin/

study.cgi?study_id=phs000178.v11.p8]. The DNA PoN is available in Google Cloud upon dbGap authorization (gs://firecloud-tcga-dcc-closed-access/reference/PoNs/final_summed_tokens.hist.bin)
The Riaz15 bulk RNA dataset used in this study is available under BioProject accession number PRJNA356761 [https://www.ncbi.nlm.nih.gov/bioproject/?term=PRJNA356761] and in the NCBI Gene Expression Omnibus (GEO) GSE91061 [https://www.ncbi.nlm.nih.gov/geo/query/acc.cgi?acc=GSE91061].
The RNA PoN is available upon dbGap GTEx approval (phs000424) (https://www.ncbi.nlm.nih.gov/projects/gap/cgi-bin/study.cgi?study_id=phs000424.v8.p2) under 'Available Phenotype and Genotype Files/Genotype Files/phg000830.v1.GTEx_WES.panel-of-normals.c1.GRU.tar'.
In addition, NCBI Human Reference Genome Build GRCh37 (hg19) was used [https://www.ncbi.nlm.nih.gov/assembly/GCF_000001405.13/].

Field-specific reporting

Please select the one below that is the best fit for your research. If you are not sure, read the appropriate sections before making your selection.

☒ Life sciences ☐ Behavioural & social sciences ☐ Ecological, evolutionary & environmental sciences

For a reference copy of the document with all sections, see nature.com/documents/nr-reporting-summary-flat.pdf

Life sciences study design

All studies must disclose on these points even when the disclosure is negative.

Sample size	We have used all available TCGA melanoma, lung adenocarcinoma and colon data with both DNA and RNA-seq.<br>We have used all samples available in Riaz et al. for which both DNA and RNA-seq were available.
Data exclusions	We have not excluded any data points.
Replication	Much of our analysis is fully deterministic. When running the ML pipeline, a SEED was set to ensure reproducibility.
Randomization	The train and test sets were randomly allocated.
Blinding	The authors were blind to the allocation of train and test sets into different groups.

Reporting for specific materials, systems and methods

We require information from authors about some types of materials, experimental systems and methods used in many studies. Here, indicate whether each material, system or method listed is relevant to your study. If you are not sure if a list item applies to your research, read the appropriate section before selecting a response.

Materials & experimental systems

n/a	Involved in the study	
☒	☐	Antibodies
☒	☐	Eukaryotic cell lines
☒	☐	Palaeontology and archaeology
☒	☐	Animals and other organisms
☒	☐	Human research participants
☒	☐	Clinical data
☒	☐	Dual use research of concern

Methods

n/a	Involved in the study	
☒	☐	ChIP-seq
☒	☐	Flow cytometry
☒	☐	MRI-based neuroimaging
